# A refined treatment strategy for skull base chordoma: A protocol and management algorithm

**DOI:** 10.1007/s10143-025-03869-4

**Published:** 2025-10-23

**Authors:** Zachary C. Gersey, Hussam Abou-Al-Shaar, David T. Fernandes Cabral, Ali A. Alattar, Hussein M. Abdallah, Michael M. McDowell, Eric W. Wang, Carl H. Snyderman, Garret Choby, Paul A. Gardner, Georgios A. Zenonos

**Affiliations:** 1 Department of Neurological Surgery, University of Pittsburgh, Pittsburgh, PA USA; 2 Department of Neurological Surgery, University of Miami Miller School of Medicine, 1475 NW 12th Ave, Miami, FL 33136 USA; 3 Department of Otolaryngology, University of Pittsburgh, Pittsburgh, PA USA; 4 Department of Neurological Surgery, Center for Cranial Base Surgery, University of Pittsburgh Medical Center, Pittsburgh, PA USA

**Keywords:** Chordoma, Skull base, Clival chordoma, Management algorithm, Radiotherapy, Genetics

## Abstract

Skull base chordomas (SBCs) are rare and challenging tumors due to their midline location and invasive behavior, requiring a multidisciplinary approach that integrates advancements in surgical techniques, radiation therapy, and emerging therapies. This paper synthesizes clinical experience with 307 surgical resections performed on 197 patients to propose a cohesive treatment paradigm for SBCs. The endoscopic endonasal approach (EEA) is highlighted as a cornerstone of surgical management, with key considerations for postoperative radiation strategies and novel therapeutic options, such as immunotherapy, discussed. By emphasizing the importance of maximal safe resection, individualized treatment planning, and ongoing innovation, this paper aims to provide a framework for optimizing outcomes in patients with SBCs while highlighting areas for future research in this complex field.

## Introduction

Chordomas are rare tumors accounting for 1–4% of bony malignancies which present a unique challenge due to their location and behavior [1, 2]. Around 38.7% originate intracranially, predominantly from the clivus. The treatment of skull base chordomas (SBCs) is inherently complex, requiring a multidisciplinary approach that combines surgical resection with adjunctive therapies such as radiation [3]. 

Skull base chordomas (SBCs) necessitate a sophisticated surgical approach [4, 5]. The endoscopic endonasal approach (EEA) is an essential technique in the management of SBCs. Over the past two decades, the EEA has undergone significant evolution with notable advancements. Advancements such as the nasoseptal flap (NSF), pituitary transposition, and far medial techniques have expanded access to these clival lesions, improving resection capabilities for challenging tumor sites [6–10]. 

Radiotherapy, primarily Proton Beam Radiotherapy (PBRT), is essential in SBC postoperative management, with options like EBRT and SRS as alternatives. While once standard after all resections, evidence now supports a more tailored approach based on surgical results and tumor genetics. Ongoing research in chemotherapy and immunotherapy, though investigational, shows promise for future chordoma treatment [11, 12]. 

In this paper, we present an expert perspective on the management of SBCs, emphasizing the integration of modern surgical techniques, advanced radiation therapy, and investigational treatments. While prior literature has described individual aspects of chordoma care—such as surgical series, radiotherapy outcomes, or molecular prognostic markers—there remains a lack of a unified, practical framework to guide decision-making across the entire disease course. By synthesizing current evidence with our institutional experience from nearly 200 patients, we aim to propose a cohesive treatment paradigm that highlights technical advances, outlines evidence-based indications for adjuvant therapies, and provides a practical algorithm to support multidisciplinary teams in optimizing outcomes for patients with SBCs. This manuscript should be understood as an expert perspective rather than a retrospective outcomes study. While we reference our institutional experience with 307 resections to provide clinical context, the primary objective is to synthesize surgical advances, radiation strategies, and emerging therapies into a unified treatment algorithm for SBCs.

## Materials and methods

This manuscript represents an expert perspective rather than a formal outcomes study. The treatment strategies described are informed by our institutional experience from 2001 to 2020 with 307 skull base chordoma resections in 197 patients. All patients had pathologically confirmed diagnoses, and all procedures were performed under protocols approved by the institutional review board (PRO11070133). These data are presented solely to provide clinical context and to illustrate the breadth of experience underlying the proposed management algorithm, rather than for statistical analysis or outcome reporting.

## Results

To provide context for the management strategies presented in this perspective, we summarize our large institutional experience. These descriptive data highlight the scope and diversity of cases that inform the proposed treatment algorithm. This cohort encompassed 197 patients, who underwent a total of 307 resections. A total of 55.8% (*n* = 110) were male and the average age at diagnosis was 43.7 years (Range 4.0–88.4). Primary tumors accounted for 43.2% (*n* = 131), where recurrences were 57.3% (*n* = 176). EEA accounted for 263 surgeries, 28 were open, and 16 were combined EEA and open. Staged surgeries accounted for 63 cases (20.5%). Among all tumors, 47.6% involved the upper clivus, 78.4% the middle clivus, 49.7% the lower clivus, 38.4% the craniocervical junction, and 14.9% were pan-clival tumors. Coronal plane extension was seen in 54.7% of cases. The average volume tumor was 21.5 cc (SD: 33.2 cc). The mean follow-up was 64.8 months (IQR 21.2–87.1 months).

## Discussion

**Fig. 1:**
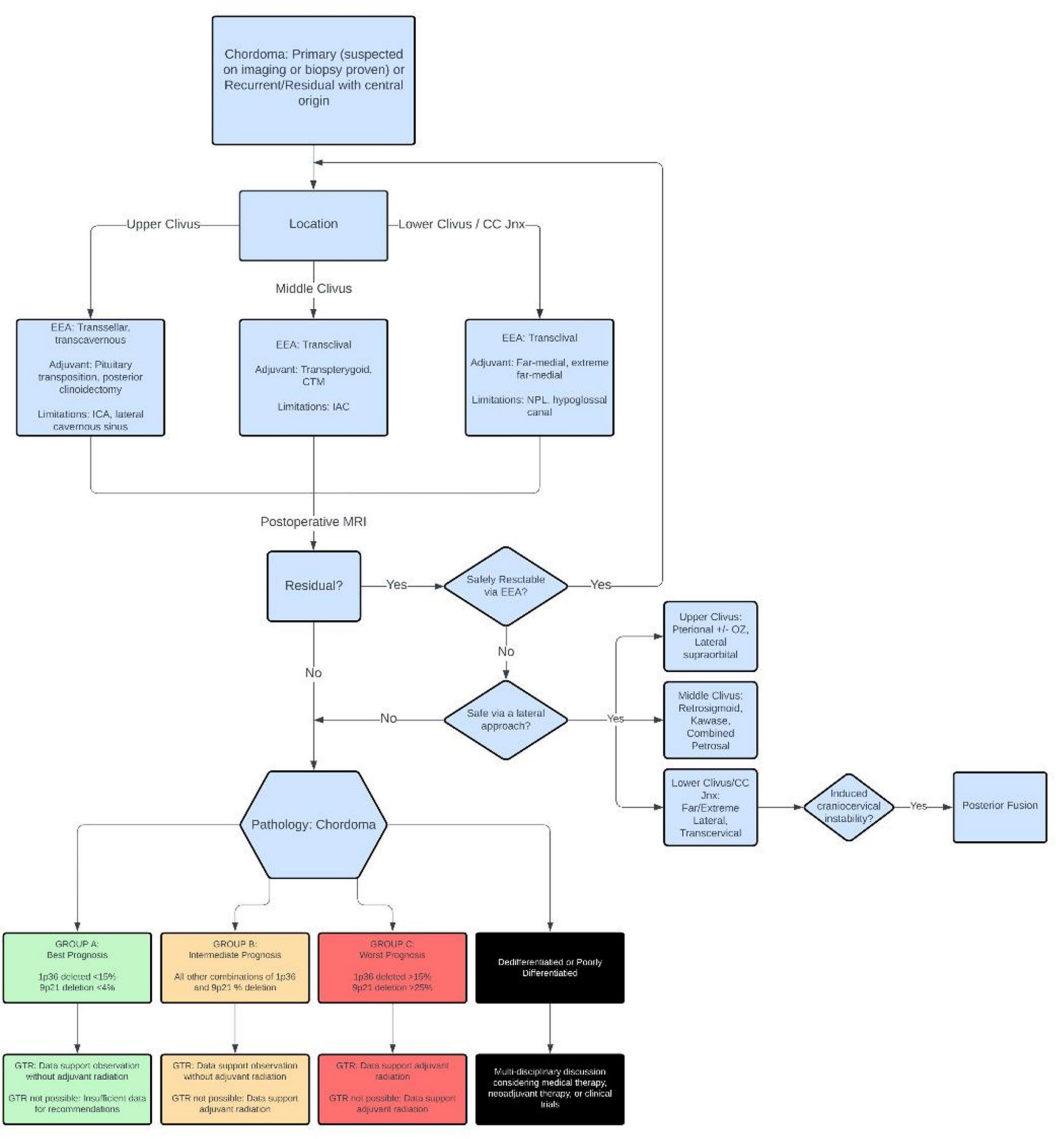
Flow-chart demonstrating the treatment protocol for skull base chordomas with central origin

In this paper, we present a detailed algorithm for the management of SBC. This comprehensive approach encompasses all stages from initial diagnosis through surgical intervention, radiation therapy, and explores potential future therapeutic strategies (Fig. 1).

## Diagnosis

The diagnosis of SBCs hinges upon a combination of clinical presentation and imaging characteristics. SBCs often present with symptoms related to mass effect on adjacent neurovascular structures. Headaches, particularly occipital, and neck pain are common presenting features occurring in 28–64% of patients, due to bony erosion and dural expansion [13]. Diplopia secondary to cranial nerve (most commonly VI) palsies are also common [14, 15]. 

MRI characteristics include low to intermediate signal intensity on T1-weighted images. T2-weighted images typically show high signal intensity, attributed to high fluid content. The enhancement pattern on contrasted MRIs varies. On CT, chordomas are often seen as well-circumscribed, expansile soft tissue masses with significant bony destruction with mild to moderate contrast uptake [16]. 

Chordomas are usually midline lesions, differentiating them from chondrosarcomas, which are eccentric. SBCs typically spread along the venous anatomy of the skull base.

## Surgical treatment

For suspected SBCs, surgical excision aims for maximal resection while preserving neurovascular function, as greater resection is linked to improved progression free survival (PFS) and overall survival (OS) [17, 18]. Standard practice involves a post-operative MRI to detect residual disease. If safely feasible, re-resection is considered during the same hospitalization, ideally before reconstruction fully heals. If a different approach is required, resection is delayed until recovery from the initial surgery.

Clival chordomas, centered medial to cranial nerves, are best accessed through ventral approaches, which minimize neurovascular manipulation. Expanded endonasal surgery offers improved resection outcomes with comparable or lower complication rates than open approaches [19, 20]. 

While lateral approaches are valuable for tumors beyond EEA reach, the EEA’s versatility and minimal invasiveness make it the primary approach for clival chordomas. Our surgical strategy is tailored to tumor size and location along clival segments: the upper clivus (posterior clinoids to sellar floor), middle clivus (sellar floor to choana roof), and lower clivus (lower skull base to foramen magnum). Craniocervical junction chordomas, extending beyond the skull base, present additional challenges.

## Upper clivus chordomas

Upper clival tumors are near critical structures like the pituitary gland, parasellar carotid arteries, and cavernous sinus. The endoscopic transsellar and transcavernous approaches, with or without pituitary transposition, are primary strategies. Larger tumors may require a suprasellar approach with pituitary transposition and posterior clinoidectomy. Interdural pituitary transposition is preferred for its versatility and lower risk of postoperative pituitary dysfunction, while posterior clinoid removal expands access to the cavernous sinus and interpeduncular cistern [10]. Extension through the roof of the cavernous sinus is approached through a trans-oculomotor triangle approach [21]. 

EEA for upper clival chordomas has limitations, especially for tumors lateral to the carotid arteries or extending into the lateral cavernous sinus, though careful medial mobilization of the parasellar carotid artery and ligation of the inferolateral trunk can provide access [22]. For tumors extending beyond the lateral cavernous wall, we use staged surgeries and lateral craniotomies, including standard pterional, orbitozygomatic osteotomies, mini-pterional, and eyebrow craniotomy.

### Middle clivus chordomas

Middle clivus chordomas require careful navigation around structures like the paraclival carotid arteries, abducens nerve, and basilar artery. The expanded EEA has proven effective for these lesions, minimizing the need for extensive traditional exposures.

The transpterygoid approach is essential in the EEA for middle clivus chordomas, involving mobilization of the pterygopalatine fossa and, occasionally, sacrificing the vidian nerve to access the medial petrous apex and Meckel’s cave [23]. Skeletonizing the paraclival carotid artery and resecting the lingual process enable lateralization of the artery, exposing the upper petrous apex. The contralateral transmaxillary (CTM) approach further extends EEA access to tumors lateral and posterior to the paraclival carotid arteries [7]. The transpterygoid and CTM approaches provide direct access to the anteromedial petrous triangle, allowing for extensive tumor resection while preserving the abducens nerve and carotid artery [24]. Notably, the CTM approach is primarily beneficial for bony lesions anterior to the abducens nerve and less effective for intradural pathology.

Despite EEA advancements, the abducens nerve remains the primary limitation and risk in these approaches, necessitating abducens nerve electromyography. The internal auditory canal (IAC) marks the lateral limit for midclival lesions. For tumors extending beyond the IAC, we supplement the EEA with retrosigmoid craniotomy, combined petrosal, or Kawase’s approach, chosen based on tumor extent to maximize safe resection and minimize neurological deficits.

## Lower clivus and craniocervical junction chordomas

Chordomas in the lower clivus and craniocervical junction pose a surgical challenge due to their proximity to critical structures like the parapharyngeal carotid and vertebral arteries, jugular tubercle, hypoglossal canal, and upper cervical vertebrae. Access is further complicated by their position at the endoscopic corridor’s inferior limit and by the intricate parapharyngeal anatomy.

To address these tumors, we utilize the far-medial and extreme medial approaches, which provide access to tumors involving the medial jugular tubercle and occipital condyle [8]. For tumors extending into the styloid and parapharyngeal spaces, eustachian tube mobilization as part of the extreme medial approach improves access [25]. 

The inferior boundary of the expanded EEA is delineated by the nasopalatine line (NPL). The NPL, extending from the anterior nasal bones to the hard palate posteriorly, predicts the lowest possible reach of the endoscopic approach [26]. Tumors extending below the NPL often necessitate a transcervical approach, used in conjunction with the EEA for comprehensive tumor resection.

The hypoglossal canal typically defines the lateral limit for lower clivus approaches. For tumors extending beyond this, a staged far-lateral or extreme-lateral approach is used, allowing effective resection while preserving vital neurovascular and skeletal structures.

## Reconstruction

Our reconstruction approach depends on the presence of an intraoperative CSF leak. For cases without meningeal violation, only a vascularized NSF is used. For posterior fossa defects with a CSF leak, we routinely apply CSF diversion based on a randomized controlled trial [27]. For high-flow CSF leaks, we use a multi-layered reconstruction with an inlay dural substitute, onlay fascia lata graft, fat graft in the clival recess to prevent pontine herniation, and an extended NSF covering the nasal floor and lateral nasal wall. For inferior clivus and craniocervical junction cases, a rhinopharyngeal flap is added, positioned against the NSF’s superior extent [28]. The viability of the NSF is routinely evaluated with ICG angiography [29]. 

**Fig. 2:**
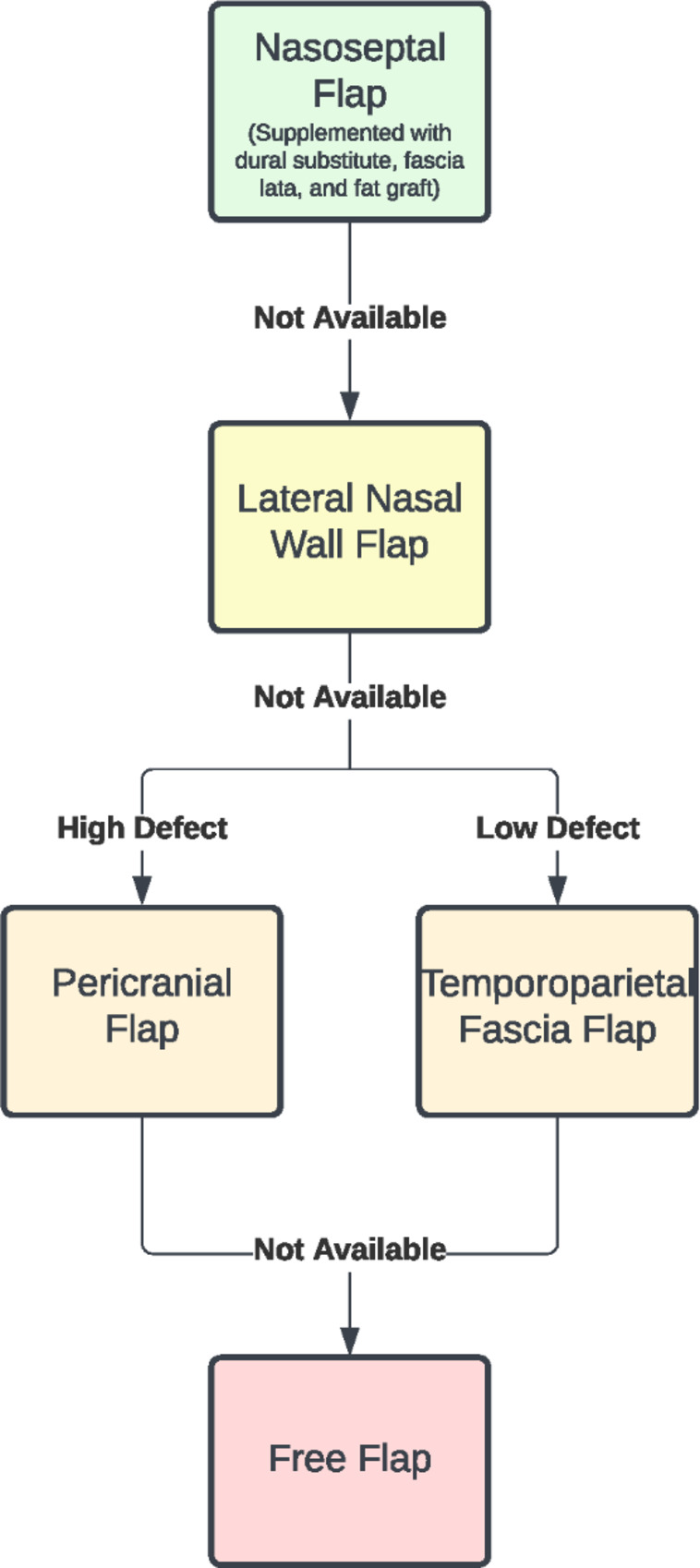
Flow-chart demonstrating the preferred order of vascularized flap options

If an NSF is unavailable due to prior surgery or pedicle injury, a lateral nasal wall flap is typically used. If this is also unavailable, a unilateral extracranial pericranial flap is employed [30, 31]. Another option is a temporoparietal fascia flap, which may have an advantage for very low defects [32, 33]. An option of last resort is a forearm or vastus free flap tunneled through the maxillary sinus [34]. The typical order of vascularized flap choice is depicted in Fig. 2.

### Extent of resection/staged surgery

The dura’s periosteal layer is typically removed with wide margins. Clival chordomas grow along the venous plexus, so the surgical strategy is tailored to these venous corridors. While wide margins are not always feasible, they are taken to aid in post-operative imaging and adjuvant radiation planning. The inner dural layer is preserved only in smaller tumors that appear free of disease.

Residual disease often occurs in the petrous apex/posterior Meckel’s cave, addressed with an anterior transpetrosal approach, and lateral to the hypoglossal canal/jugular foramen, managed with a far-lateral approach. Craniocervical junction chordomas extending into Batson’s plexus below C2 typically require a posterior or trans-cervical approach.

We combine approaches as needed to achieve maximal safe tumor resection, based on the clival segment involved. However, GTR is never prioritized over preserving function or vital structures like the vertebral artery or a potentially recoverable abducens nerve.

### Postoperative radiation

Due to their complex neurovascular involvement and resistance to conventional chemotherapy, SBCs require postoperative radiation as a key management element. However, the optimal timing, modality, dosage, and protocols for radiotherapy remain debated and vary across centers [35]. 

Advances in genomic profiling have informed our approach to postoperative radiation. Genotypic grouping based on 1p36 and 9p21 deletions guides treatment: Group A tumors (low 1p36 deletion and homozygous 9p21 deletion) generally have a better prognosis and may need less radiation, especially after GTR. In contrast, Group B and C tumors without complete resection, show poorer PFS and benefit more from radiation [36, 37]. 

Due to chordomas’ relative radioresistance, high radiation doses are often required, with EBRT historically delivered up to 75 Gy[. Proton beam radiation therapy (PBRT) is increasingly preferred for its superior dose distribution and reduced impact on surrounding neurovascular structures. PBRT is the recommended modality for postoperative radiation, while stereotactic radiosurgery is a viable alternative for patients with inoperable focal recurrences.

### Surveillance

Patients are followed postoperatively with clinical exams and MRIs. We recommend an MRI immediately after surgery, then at three and nine months. If no progression or recurrence is observed, annual MRIs are advised, with more frequent scans for dedifferentiated or poorly differentiated tumors requiring closer monitoring [16]. A craniospinal MRI is routinely performed at initial diagnosis to rule out distant metastases.

### Recurrences

Managing recurrent SBC is complex and requires clinical judgment. For focal recurrences that are surgically accessible, especially with new neurological deficits, we recommend repeat excision, contingent on the patient’s health and surgical tolerance.

For focal recurrences that are not amenable to surgical intervention due to either their location or the patient’s inability to undergo surgery, we advise considering stereotactic radiosurgery as an alternative treatment option [38]. This technique allows for precise targeting of the tumor while minimizing damage to surrounding healthy tissue, making it a suitable option for inoperable cases.

In scenarios where the recurrence is diffuse, widespread, or has occurred multiple times, and where surgery is not a viable option, we recommend consultation with an oncologist to explore the possibility of enrolling in a clinical trial. This approach is particularly pertinent given the lack of standardized chemotherapy regimens for chordoma. Clinical trials may provide access to novel therapeutic agents or strategies that are not yet widely available.

### Chemotherapy/Immunotherapy

While no chemotherapy agents have been FDA-approved specifically for chordoma, ongoing research into the genetic and molecular characteristics of skull base chordomas is identifying potential targets for future treatments. Central to these discoveries is brachyury, a transcription factor recognized as a crucial molecular driver in chordoma development [39]. Various clinical trials are investigating small-molecule inhibitors and vaccines aimed at targeting brachyury. However, to date, these trials have yet to demonstrate a significant positive impact in patients with chordoma (Clinical trial: NCT03083678) [40]. 

Immunotherapy shows promise for chordoma treatment due to high PD-L1 expression in these tumors. Ongoing trials with checkpoint inhibitors like Nivolumab and Pembrolizumab have shown mixed results [41]. Given current evidence, our institution does not endorse a specific chemotherapy or immunotherapy protocol for SBCs. We hope ongoing research will lead to more effective treatment options in this challenging field.

In cases of biopsy proven poorly differentiated or dedifferentiated chordoma subsequent steps in management often involve a multidisciplinary panel considering chemotherapy or targeted therapy as neoadjuvant options.

### Study limitations

This study has several limitations that warrant consideration. As a single-institution, retrospective experience, the findings may be subject to selection bias and may not be fully generalizable to other centers. Additionally, while we present a large surgical experience, the primary intent of this manuscript is to provide a practical framework and guide for surgeons and physicians managing skull base chordomas. For this reason, detailed outcome analyses were not included, as such data are more appropriately addressed in future, dedicated outcome-focused studies.

## Conclusion

Managing skull base chordomas requires a multidisciplinary approach. The expanded EEA enables maximal resection while preserving neurovascular structures. For recurrences, strategies range from re-excision to stereotactic radiosurgery, depending on tumor accessibility and patient status. PBRT remains essential due to chordomas’ radioresistance, with genomic profiling guiding radiation decisions. Ongoing research into chemotherapy and immunotherapy, particularly targeting molecular drivers, offers hope for advancing treatment. These findings highlight our commitment to improving SBC outcomes through clinical expertise and innovation.

## Data Availability

No datasets were generated or analysed during the current study.

## Abbreviations

SBC: Skull Base Chordoma
EEA: Endoscopic Endonasal Approach
NSF: Nasoseptal Flap
PBRT: Proton Beam Radiotherapy
EBRT: External Beam Radiotherapy
SRS: Stereotactic Radiosurgery
PFS: Progression-Free Survival
OS: Overall Survival
IQR: Interquartile Range
CTM: Contralateral Transmaxillary (approach)
MRI: Magnetic Resonance Imaging
CSF: Cerebrospinal Fluid
NPL: Nasopalatine Line
ICG: Indocyanine Green
IRB: Institutional Review Board
FDA: Food and Drug Administration
